# Genetic Patterns of Domestication in Pigeonpea (*Cajanus cajan* (L.) Millsp.) and Wild *Cajanus* Relatives

**DOI:** 10.1371/journal.pone.0039563

**Published:** 2012-06-22

**Authors:** Mulualem T. Kassa, R. Varma Penmetsa, Noelia Carrasquilla-Garcia, Birinchi K. Sarma, Subhojit Datta, Hari D. Upadhyaya, Rajeev K. Varshney, Eric J. B. von Wettberg, Douglas R. Cook

**Affiliations:** 1 Department of Plant Pathology, University of California Davis, Davis, California, United States of America; 2 Department of Mycology and Plant Pathology, Banaras Hindu University, Varanasi, India; 3 Indian Institute of Pulses Research, Uttar Pradesh, India; 4 International Crops Research Institute for the Semi-Arid Tropics, Patancheru, Andhra Pradesh, India; 5 Department of Biological Sciences, Florida International University, Miami, Florida, United States of America; 6 Center for Tropical Plant Conservation, Fairchild Tropical Botanic Garden, Coral Gables, Florida, United States of America; Michigan State University, United States of America

## Abstract

Pigeonpea (*Cajanus cajan*) is an annual or short-lived perennial food legume of acute regional importance, providing significant protein to the human diet in less developed regions of Asia and Africa. Due to its narrow genetic base, pigeonpea improvement is increasingly reliant on introgression of valuable traits from wild forms, a practice that would benefit from knowledge of its domestication history and relationships to wild species. Here we use 752 single nucleotide polymorphisms (SNPs) derived from 670 low copy orthologous genes to clarify the evolutionary history of pigeonpea (79 accessions) and its wild relatives (31 accessions). We identified three well-supported lineages that are geographically clustered and congruent with previous nuclear and plastid sequence-based phylogenies. Among all species analyzed *Cajanus cajanifolius* is the most probable progenitor of cultivated pigeonpea. Multiple lines of evidence suggest recent gene flow between cultivated and non-cultivated forms, as well as historical gene flow between diverged but sympatric species. Evidence supports that primary domestication occurred in India, with a second and more recent nested population bottleneck focused in tropical regions that is the likely consequence of pigeonpea breeding. We find abundant allelic variation and genetic diversity among the wild relatives, with the exception of wild species from Australia for which we report a third bottleneck unrelated to domestication within India. Domesticated *C. cajan* possess 75% less allelic diversity than the progenitor clade of wild Indian species, indicating a severe “domestication bottleneck” during pigeonpea domestication.

## Introduction

One common feature of domesticated organisms is reduced genetic diversity compared to their wild relatives. Two major forces that cause the reduction in genetic diversity are small population sizes (“founder effect”) that occur during the initial formation of a domesticated lineage, and selective sweeps and/or directional selection for genes associated with domestication traits [1]. Intensive breeding, which is a recent phenomenon relative to domestication, typically causes further reductions to genetic diversity [2] and understanding such shifts at the molecular genetic level can inform crop improvement programs. Although the impact of such processes on genetic diversity are reasonably well described for major crops such as maize, wheat, soybean and rice [3]–[7], for many minor crops, which are often of significant regional importance, the circumstances of domestication are poorly described.

As much as domestication is a human-driven process, it can also be influenced by random gene flow from wild relatives. Many crops, particularly minor crops of regional importance, are still grown alongside their wild relatives, increasing the opportunity for gene flow between cultivated and non-cultivated populations. Although such gene flow reduces our ability to characterize domestication-related processes, its occurrence over protracted periods can allow for the contribution of novel traits from locally-adapted wild populations of related species into domesticated forms [8]. Geographical and/or environmental factors can also constrain genetic change during domestication. For example, in cases where recent (i.e., post-Columbian) expansion of minor crops has taken cultivated genotypes beyond areas of their historical domestication, analyses of genetic diversity may reveal bottlenecks and nested patterns of domestication that reflect new populations adapting to new environments or regional human preferences.

Pigeonpea (*Cajanus cajan* (L.) Millsp.) is a widely adapted, drought tolerant food legume crop cultivated throughout the semi arid tropics and subtropics. Though considered a minor crop, pigeonpea is of considerable importance in areas of South Asia (mainly on the Indian-subcontinent), Africa, the Caribbean and Latin America, where it is a prominent source of protein nitrogen in the human diet, as well as wood for fuel and light duty structural applications such as thatch for roofing. Grown on 4.63 million hectares, pigeonpea ranks 6^th^ among grain legumes in production [9]. The genus *Cajanus* is composed of 34 species [10], among which pigeonpea is the only cultivated member, with the remaining wild relatives assigned to the secondary or tertiary gene pools according to the gene pool concept of Harlan and de Wet [11]. Hybridization is widespread in the genus and many wild species can be crossed to cultivated *C. cajan*, a feature that has enabled the use of inter-specific crosses in breeding programs [12]–[13]. The majority of *Cajanus* species are endemic and confined either to Southern/South-Eastern Asia or Australia [14], [15]. Given this substantial overlap in geographic distribution and the high degree of cross-compatibility among species, it seems probable that many *Cajanus* species are parts of species complexes that arose through current or recent natural gene flow.

Morphological evidence suggests that *C. cajanifolius*, which is native to the Indian subcontinent, is the progenitor of pigeonpea [16], [17]. Nevertheless, historians and scientists have debated the center of origin for pigeonpea, with arguments in favor of either an African [18], [19] or Indian [16], [17], [20] origin. Proponents of the African center of origin typically cite the presence of a single endemic wild species (*C. kerstingii*) in Africa [18], [19]. The bulk of evidence, however, favors an Indian origin, with some authors postulating a Northern Indian origin no earlier than 3,500 years ago [16], [17], [20]. Evidence in favor of an Indian origin includes the presence, in India, of several wild, morphologically diverse species including the putative wild progenitor (*C. cajanifolius*), as well as archaeological remains, linguistic evidence, and a variety of uses in the daily cuisine within India [17]. Archeological records reveal pigeonpea seeds in Maharashtra, a State in India, from the 2nd century BC to the 3rd century BC [21]. The proposed route of dispersion of pigeonpea from India is to Malaysia and East Africa, on to West Africa and finally to the West Indies where it was named pigeonpea in 1962. Pigeonpea arrived in the New World through the slave trade from Africa [22].

Pigeonpea germplasm represents a diverse set of landraces and heterogeneous feral forms that are adapted to various agro-ecological settings [23]. Despite extensive phenotypic diversity, molecular evidence from Simple Sequence Repeats (SSRs) [24] and Diversity Array Technology (DArT) [25] suggests very low genetic diversity within cultivated pigeonpea when compared to its wild relatives. The only means to broaden the genetic base of domesticated *C. cajan* is to introgress genetic diversity from the wild gene pool [23], and thus understanding how diversity is assorted among pigeonpea and its wild relatives has practical implications.

With the objective of understanding genetic diversity among *Cajanus* species and inferring patterns of domestication, we examined allelic variation in domesticated pigeonpea and its wild relatives using a set of gene-based single nucleotide polymorphisms. The genetic signatures of domestication that we identify suggest a primary bottleneck within subtropical India, the likely center of domestication, and a nested bottleneck associated with pigeonpea that is cultivated in disperse tropical regions, which we speculate is the consequence of breeding for adaptation to a new environment. Moreover, we provide evidence of both modern and archaic gene flow between pigeonpea and wild relatives, including a third genetic bottleneck in Australian *Cajanus* species that is unrelated to the India-centric domestication of modern pigeonpea.

## Results

Single nucleotide polymorphisms (SNPs) were assayed in a total of 110 accessions representing cultivated *C. cajan* (79 accessions) and its wild relative relatives (31 accessions) (Table S1), all of which belong to the genus *Cajanus*. The wild accessions represent 13 of 34 known species, while the cultivated group includes modern varieties, pre-breeding material, land races, as well as perennial pigeonpea accessions obtained from non-agricultural settings that are presumed to be feral forms. These genotypes originate from widespread geographical regions, spanning the known distribution of *Cajanus* species and represent both tropical and subtropical environments of Africa, Asia, Latin America, the Caribbean, the Indian sub-continent and Australia.

Individual SNPs were identified based on comparisons of sequences of *C. cajan* accession ICP 28 and *C. scarabaeoides* accession ICPW 94 in a set of low-copy orthologous genes. These two species span an evolutionary distance that is wider than the proposed domestication gradient from cultivated *C. cajan* to its presumed progenitor *C. cajanifolius*. Excluding failed sequencing reactions and monomorphic loci, single nucleotide polymorphisms were identified in a total of 670 unique genes from which 752 SNPs were used to design a GoldenGate genotyping assay (Table S2). Within this set of nucleotide variants, a minimum of 16.6% represent shared ancestral variation, while 36.8% and 68.2% of loci were polymorphic within *C. scarabaeoides* and/or the remaining *Cajanus* species group, respectively.

### Phylogenetic Relationships between Wild and Domesticated Groups

**Figure 1:**
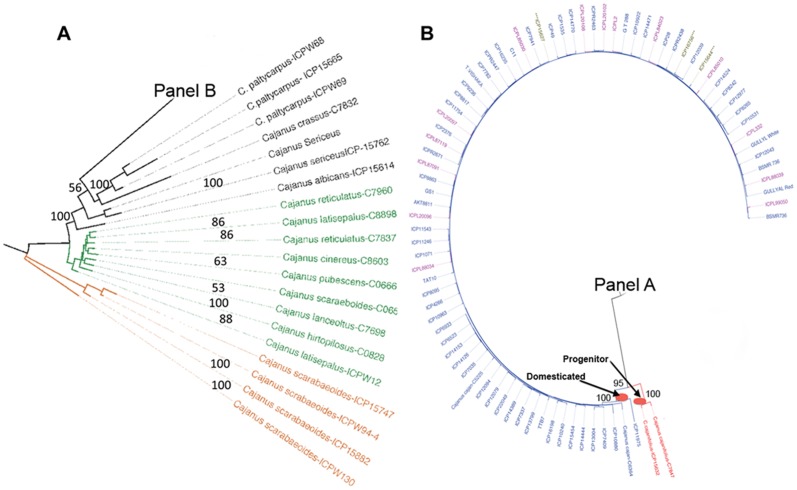
Neighbor Joining phylograms depicting wild and domesticated pigeonpea. Panel **A**, relationships among wild Cajanus species. Species groups are designated by color: orange, Wild*-Scarabaeoides*; green, Wild-Australia; black, Wild-India. Panel **B**, expansion of domesticated lineages from Panel A. The nature of accessions is reflected in their colors: red, wild progenitor; blue, cultivars and genebank accessions; pink, landraces. *** indicates mislabeled accessions annotated as wild species. The two Neighbor-Joining trees are linked to each other at Panel B and Panel A respectively. Bootstrap values of ≥50% are shown above their respective branches.

**Figure 2:**
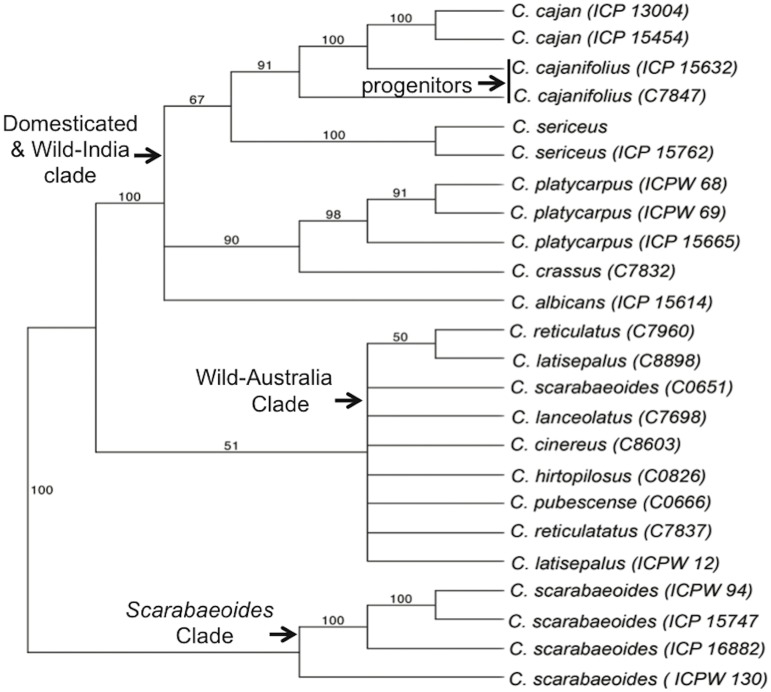
Phylogeny of *Cajanus* species depicted as a 50% majority rule consensus tree. Tree topology was inferred with maximum parsimony via heuristic searches among 1000 trees. Numbers above branches indicate bootstrap support (>50%). The vertical bar indicates the putative projentitor species (two *C. Cajanifolius* accessions). Tree length  = 2145, consistency index (CI)  = 0.638, and retention index (RI)  = 0.837. The Bootstrap support for the sister relationship between wild-Australia and Wild-India clades (in the absence of the two *C. cajan* accessions) is 100% (data not shown).

The combined data set was used to calculate pair-wise distances between all genotypes and thereby generate a dissimilarity matrix from which a weighted Neighbor Joining tree was calculated. Previous analysis of nuclear *ITS* and chloroplast *trnL-F* spacer sequences suggested that *C. scarabaeoides* is the most basal member of the *Cajanus* clade (MT. Kassa, PhD dissertation) and thus *C. scarabaeoides* was used to root the Neighbor Joining tree. Similarly, a parsimony phylogenetic analysis was performed using all wild accessions and two representative samples of the domesticated (*C. cajan*) accessions. Both parsimony and Neighbor Joining trees resolved congruent topology with overall similarity on major clades (Figures 1 and 2). Three well-resolved clades were evident from this analysis, including a basal set of *C. scarabaeoides* of Indian origin, and two sister clades representing wild species of Australian origin and a more diverse but well-supported clade containing the remaining wild *Cajanus* species that were exclusively of Indian origin (Figures 1 and 2).

The tree topology in Figures 1 and 2 reflects both the distinctiveness of species and the geographical origin of species’ groups. Thus, domesticated accessions formed a monophyletic group that was internal to, and significantly less diverse than, the group of non-*scarabaeoides* wild species of Indian origin. *C. cajanifolius* has been nominated as the progenitor of domesticated pigeonpea based largely on morphometric and alpha-taxonomic criteria [17], [20]. Indeed, two accessions of *C. cajanifolius* (C7847 and ICP 15632) are sister to the large group of domesticated genotypes in maximum parsimony analysis with strong bootstrap support (91%), validating *C. cajanifolius* as the most recent progenitor for pigeonpea. These *C. cajanifolius* accessions, which we speculate are true wild representatives, are closely related to the wild non-*scarabaeoides* species of Indian origin with the expected affinities to coherent sets of *C. platycarpus* and *C. sericeus* genotypes, as well as to individual representatives of *C. albicans* and *C. crassus*.

Phylogenetic tools are not suited to analysis of individual genotypes with strongly reticulate histories, as is the case with genotypes from a single species or breeding pool. Nevertheless, Neighbor Joining analysis does identify similarity among sets of genotypes (see Figure 1) and many of these similarities are congruent with the known history of individual accessions and supported by subsequent population genetic analyses (see below). Thus, *C. cajan* C6364 and ICP 11975 were the most basal genotypes among the domesticated accessions. Interestingly, *C. cajan* C6364 is annotated as a naturally occurring, semi-domesticated and rarely found Australian woody herbaceous pigeonpea, while ICP 11975 is a genotype from the Philippines. The distant relationship of *C. cajan* C6364 to other *Cajanus* spp. of Australian origin, and its close affinity with domesticated *C. cajan*, is consistent with the origin of *C. cajan* C6364 as a feral genotype, and in fact both *C. cajan* C6364 and ICP 11975 show evidence of genetic admixture (see below). The data also suggest that at least three accessions are misclassified, as they are annotated as wild non-*cajan* species (i.e., ICP 15627, *C. albicans*; ICP 15756, *C. scarabaeoides*, and ICP 15644, *C. lineatus*) but were well integrated into the domesticated group.

**Figure 3:**
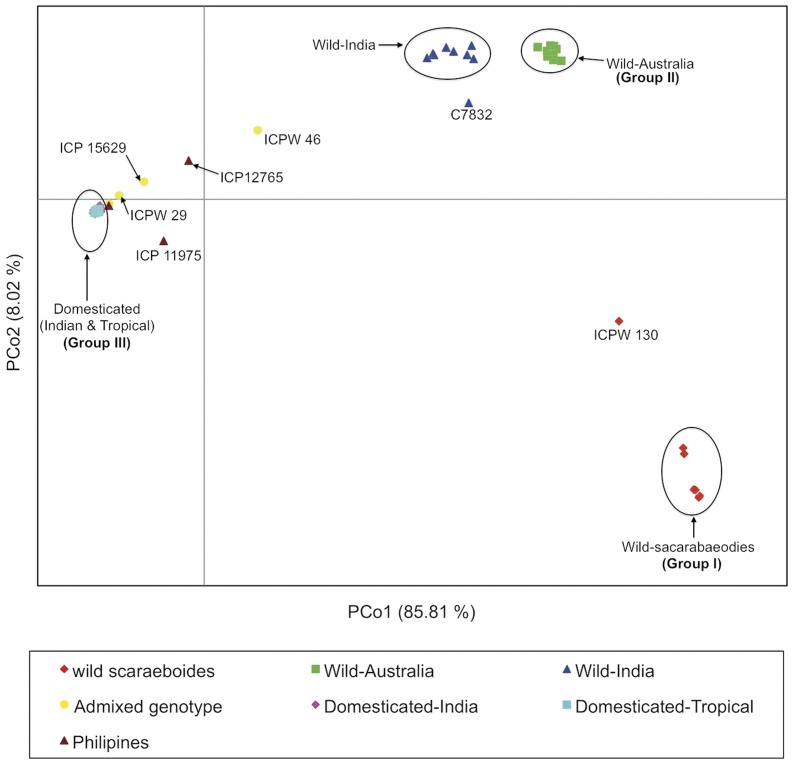
Principal coordinates analysis of domesticated pigeonpea and wild relatives. Red diamonds, wild *C. scarabaeoides*; green squares, wild-Australia *Cajanus* spp; dark blue triangles, wild-India *Cajanus* spp; light blue squares and pink diamonds represent domesticated *C. cajan* of tropical and Indian origin, respectively. Admixed genotypes between domesticated and wild species are labeled with yellow circles, or in the case of admixed Philippines accessions with dark red triangles. Accession numbers were added for accessions mentioned in the text.

To further assess relationships among accessions we conducted a Principal Coordinate Analysis (PCoA) using GenAlEx v.6.3 [26]. This multivariate approach was chosen to complement phylogenetic analysis because phylogenetic analyses are more sensitive to relationships between related individuals whereas PCoA is more informative regarding distances among major groups [27]. Principal Coordinate Analysis (PCoA) distinguished three groups of individuals (I, II and III) along discriminate axes 1 and 2, which accounted for 85.81% and 8.02% of the genetic variation, respectively (Figure 3). Along the first axis, wild accessions were resolved from domesticated accessions, while the second axis resolved the Indian *C. scarabaeoides* group (group I) from the remaining wild accessions of both Australian and Indian origin (group II). Within group II the Australian set forms a homogenous subgroup and the Indian genotypes form a more diverse assemblage, consistent with the previous phylogenetic analysis. Group III contained the domesticated *C. cajan* cluster. The low level of variation of the domesticated cluster is reflected in the tight clustering of most genotypes. Interestingly, ICP 11975 is an outlier from the main domestication group in Figure 3, supporting its basal affiliation to the domestication lineage predicted by Neighbor Joining analysis (Figure 1). A single accession of *C. cajan* from the Philippines (ICP 12765), as well as *C. cajanifolius* accessions ICPW 29 and ICP 15629, and *C. lineatus* ICPW 46, were also outliers in the PCoA analysis (Figure 3). Analysis of allele frequencies (see below) suggested a high proportion of genetic admixture for these genotypes and we suggest that these accessions originated as hybrids between wild and cultivated forms.

### Genetic Structure of Wild and Domesticated Pigeonpea

To investigate genetic relationships among accessions and to search for evidence of genetic admixture between cultivated and wild genotypes, we utilized the Bayesian algorithm STRUCTURE [28], [29]. STRUCTURE uses allele frequencies to derive subsets from a set of sampled individuals that approximate Hardy-Weinberg equilibrium, and thus represent subpopulations in the genetic sense. In the current study the taxonomic divisions are species level distinctions and, with the exception of *C. cajan*, sampling of multiple accessions within a species was limited. Thus, the population genetic processes that STRUCTURE is sensitive to occur within the cultivated group of accessions, but not the more basal groups of diverged species. Nevertheless, in the combined analysis STRUCTURE served to delimit the primary subdivisions circumscribed by phylogenetic analysis and provided the basis to investigate the possibility of gene flow between groups.

**Figure 4:**
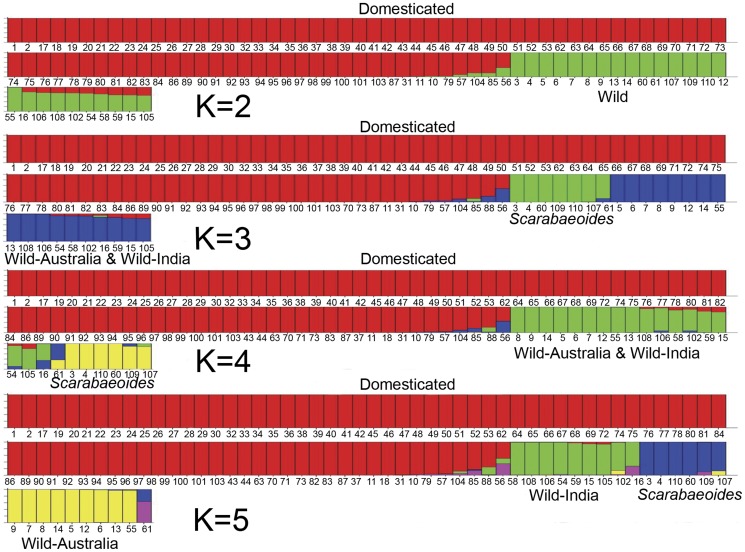
Population structure among the wild and domesticated genotypes of *Cajanus*. Output of the population genetic program STRUCTURE at increasing K values of 2 to 5. The primary divisions at increasing K values mirror phylogenetic groupings shown in Figures 1 and 2. Memberships of individual genotypes to specific subgroupings are indicated by colored bars. Genetic admixture is evidenced by fractional membership in multiple subgroups. The correspondence between numbers below each genotype and specific accessions is given in Table S1. Likelihood values for each value of K are given in Table S3.

When one has knowledge of the biology and history of a set of accessions, analyzing the partitioning of accessions into “K” subgroups can be informative (Figure 4 and Table S3). At optimal K = 3 domesticated accessions were resolved from a second group of wild accessions from India and Australia, and from a third group of *C. scarabaeoides* accessions, congruent with previous phylogenetic and Principal Coordinate Analysis (PCoA) (Figures 1, 2 and 3). At K = 2 STRUCTURE distinguished the wild and domesticated groups, mirroring axis one of the PCoA analysis (Figure 3), and only at K = 5 were the major phylogenetic lineages shown in Figures 1 and 2 well resolved. At K values of 2, 3 and 4 the wild species of Indian origin consistently shared partial membership (12–17%) with the domesticated group, although their primary membership was with wild Australia. This shared membership is not unexpected given the hypothesis that domesticated *C. cajan* is derived from the wild India group, and indeed among 209 loci reporting shared membership 36% of SNP were common to a majority (>7 of 9) of wild India accessions. By contrast, 12% of these 209 SNPs were shared between domesticated accessions and only one of the wild India species; for example, half of this set (6% total) was associated only with *C. crassus*, the most basal of wild-India species. This observation is consistent with admixture between domesticated accessions and *C. crassus* and raises the possibility of significant rates of gene flow between domesticated lineages and other species within the wild-India clade.

**Figure 5:**
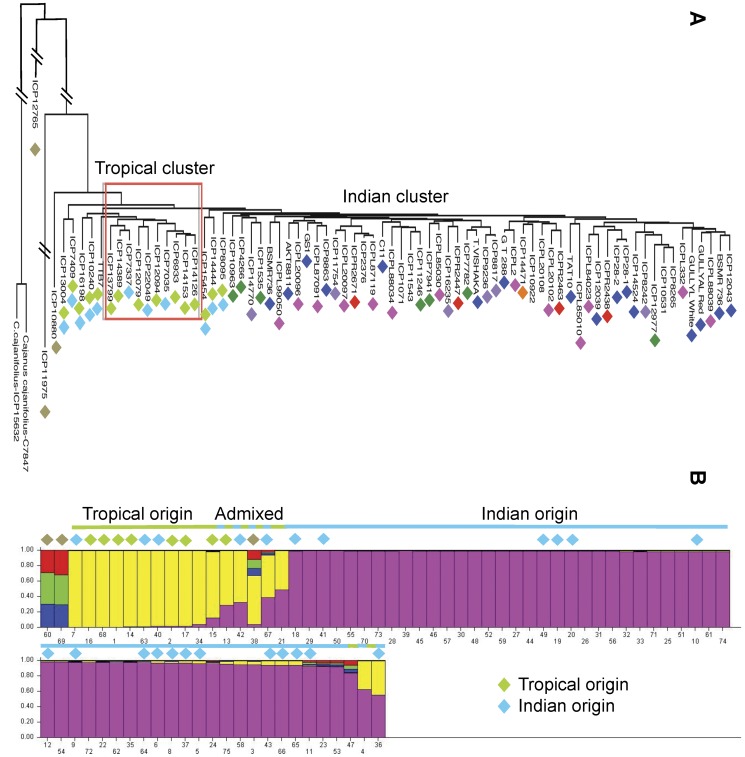
Population structure of cultivated *Cajanus cajan*. Panel **A**, Weighted Neighbor-Joining tree depicting pairwise relationships between accessions. Colors denote the nature of individual accessions: Blue diamonds, cultivars and elite varieties; Pink diamonds, landraces; purple diamonds, ICRISAT reference material; Green diamonds, Core collection; Red diamonds, R-line; Orange diamonds, Minicore; Light green diamonds, Tropical; light blue, Indian; Light brown diamonds, Philipines. Genotypes with admixture between Indian and Tropical subgroups are designated by both light green and light blue diamonds. Panel **B**, population subdivisions with cultivated genotypes revealed by STRUCTURE. Green diamonds, genotypes with tropical distribution; blue diamonds, genotypes with an Indian sub-tropical distribution. Admixed genotypes are those with fractional membership in multiple groups. Likelihood values for each value of K are given in Table S4.

**Figure 6:**
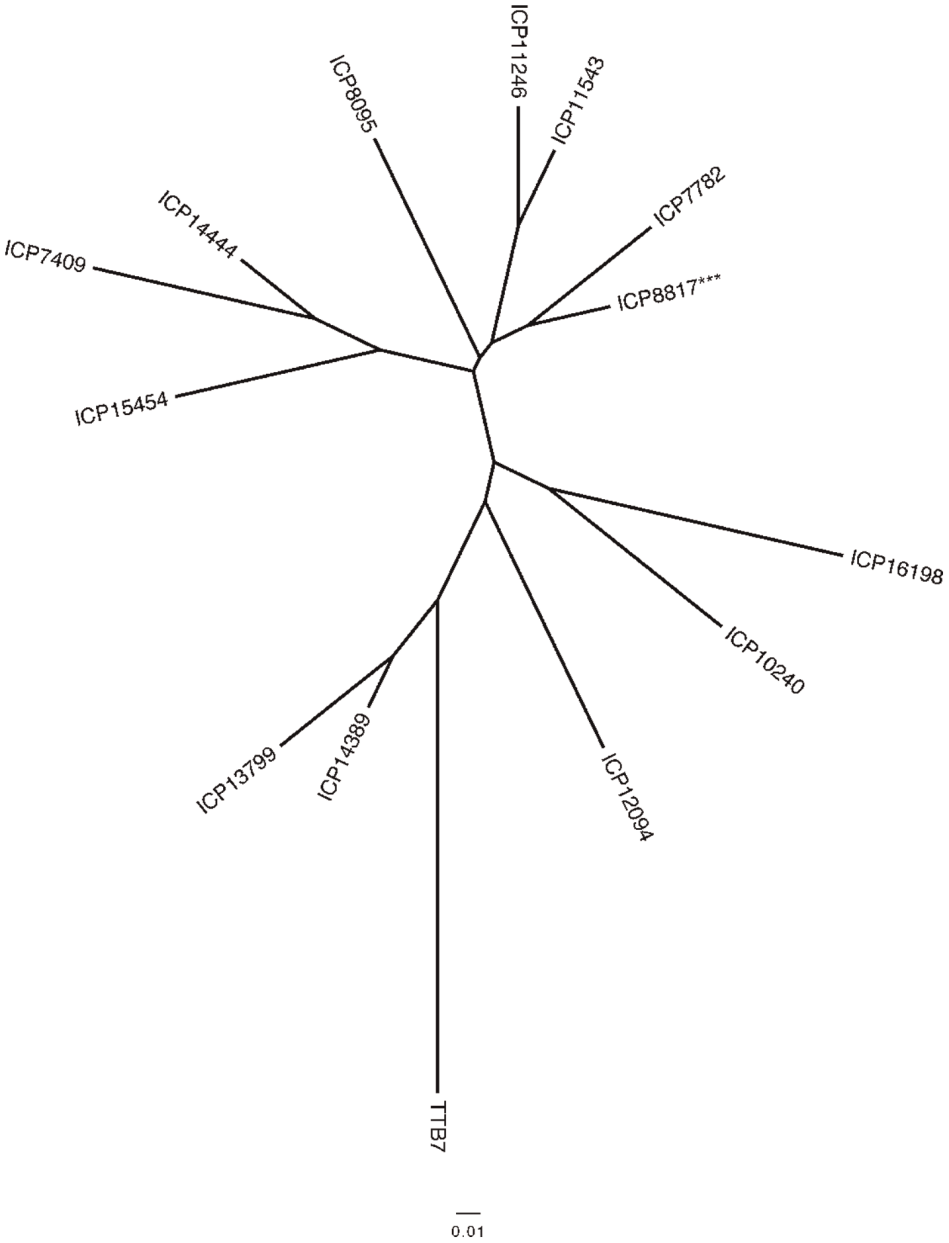
Inferred relationships among admixed *Cajanus cajan* accessions. An un-rooted Neighbor Joining tree depicting pairwise similarity between genotypes with simple admixture between domesticated-India and domesticated-tropical accessions. Admixed genotypes were identified based on mixed-group membership defined in Figure 5B. Accession ICP 8817 has an identical haplogype to accession ICP 9236, and thus only ICP 8817 is shown.

We analyzed the cultivated accessions by themselves to determine if genetic structure could be detected without the confounding effect of far greater differences among the wild taxa. As shown in Figures 5 and Table S4, the results indicate a predominant genetic subdivision that mirrors the eco-geographic history of the associated genotypes, with one group containing all accessions from tropical regions in Africa, the Caribbean, and Latin America, and the second group containing genotypes exclusively of Indian origin. Thirty percent of “domesticated” accessions showed evidence of genetic admixture between Indian and tropical accessions (Figure 5B). Analysis of admixed SNPs revealed examples of simple admixture between the tropical and Indian groups and complex patterns of admixture involving alleles from the wild-India lineage. When considered together, genotypes with complex patterns of admixture contained 165 segregating SNPs, of which 76% occurred only in one admixed genotype. These divergent SNP patterns are consistent with the origin of these genotypes through independent hybridization events rather than common ancestry. Genotypes with complex patterns of admixture include three genotypes from the Philippines, including those previously inferred as being admixed based on Neighbor Joining and PCoA analyses, and five additional *C. cajan* accessions of Indian origin (ICP 12977, TAT10, ICPL 85010, ICPL 99050 and ICP 13004). Fifty-eight SNPs (29 loci) among 15 cultivated accessions were implicated in the simple admixture. Figure 6 depicts a proposed network of relationships among these individuals that is suggestive of breeding history, though we stress that deeper genotyping is necessary to fully validate these inferences.

Only in the case of *C. cajan* ICP 11975 was there evidence of genetic contribution from the *C. scarabaeoides* gene pool to a domesticated genetic background. This is interesting given the status of ICP 11975 as a Philippines “*C. cajan*” accession. ICP 11975 was also unusual as an outlier in the PCoA analysis (Figure 3), reflecting its unique genetic constitution. *C. cajanifolius* ICPW 29 and ICP 15629 had >95% membership in the domesticated cluster, with less than 5% contribution from the wild background, and thus would be more appropriately referred to as admixed accessions of *C. cajan*, rather than as accessions of *C. cajanifolius*. *C. lineatus* (ICPW 46) was admixed in nearly equal proportions from both wild and domesticated backgrounds, and had been previously proposed as an admixed genotype based on morphology by van der Maesen (personal communication).

### Genetic Variation among Wild and Domesticated Accessions

To find groups with reduced genetic variation, we used Analysis of Molecular Variance (AMOVA) to partition variance among hierarchical sets of individual genotypes (three groups and six populations) that were circumscribed by a combination of phylogenetic analysis, geographical origin, breeding history, and the outputs of PCoA and STRUCTURE: Group I, wild-*scarabaeoides*; Group II, wild-Australia and wild-India; Group III, domesticated-India, domesticated-tropical, and domesticated Philippines. As shown in Table 1, most genetic variation was attributed to differences among the three groups (89%), although genetic differentiation was evident at all levels of analysis. These broad patterns of genetic differentiation reflect patterns established in previous phylogenetic, PCoA and STRUCTURE analyses.

**Table 1:** Summary results of AMOVA analyses within and among populations of 95 accessions of the domesticated and wild groups.

Source	df	SS	MS	Est. Var.	% Variation	Statistics	Value	P
**Among groups**	2	18135.809	9067.905	492.714	**89%**	**Fct**	**0.895**	**0.010**
**Among pops within groups**	3	1107.072	369.024	30.265	**5%**	**Fsc**	**0.521**	**0.010**
**Among Individuals within pops**	89	2476.214	27.823	27.823	**5%**	**Fst**	**0.949**	**0.010**
**Total**	94	21719.095		550.801	100%			

d.f.: Degrees of freedom; SS, sum of squared observations; MS, mean of squared observations; Est. var., estimated variance % Var., percentage of total variance; Fct, proportion of the total genetic variance between groups; FSc, proportion of the total genetic variance among populations within a group; Fst, proportion of the total genetic variance within populations.

Genetic polymorphism was highest within wild groups of Indian origin (both wild-India and wild-*scarabaeoides*), with rates ∼3-fold higher than within the wild-Australian group (∼37% polymorphism compared to ∼12%) (Table 2). In contrast, the lowest rates of polymorphism were documented for the domesticated populations, with the domesticated-tropical population having ∼60% the polymorphism of the domesticated-India population and no private alleles relative to the domesticated-Indian group. As expected, there was little genetic differentiation between domesticated-India and domesticated-Tropical populations (Fst = 0.05). In contrast, the Philippines accessions showed relatively high genetic differentiation when compared to these domesticated groups (Fst = 0.179) and possessed overall high levels of polymorphism, consistent with our previous suggestion of independent admixture events leading to these individual accessions.

**Table 2:** Percentage of Polymorphic loci in wild and domesticated groups.

Sub-groups	Number of accessions	Genetic Status	Polymorphic loci (%)
**Wild** ***scarabaeoides***	4	Wild	36.7%
**Wild** **Australian**	9	Wild	11.84%
**Wild** **Indian**	9	Wild	37.37%
**Domesticated Indian**	58	Domesticated	8.64%
**Domesticated** **Tropical**	12	Domesticated	5.45%
**Philippines**	3	Domesticated	23.94%
**Mean**			**20.66%**
**SE**			**5.78%**

Domesticated populations were considerably less differentiated from the wild-Indian population relative to differentiation from either wild-Australia or wild-*scarabaeoides* (Table 3). Even lower genetic differentiation was observed between wild-India and wild-Australia (Fst = 0.290), which is consistent with phylogenetic and allele frequency analyses that establish these populations as sister groups. In contrast to the low genetic distance (Table 4) with wild-India, wild-Australia was strongly differentiated from the domesticated groups, suggesting that the divergence between wild-India and wild-Australia was archaic relative to domestication of *C. cajanifolius* from wild-India, or that domestication occurred from an isolated subpopulation within the wild-India group.

**Table 3:** Pairwise estimates of FST among wild and domesticated groups.

	Wild *scarabaeoides*	Wild Australian	Wild Indian	Domesticated Indian	Domesticated Tropical	Philippines
**Wild** ***scarabaeoides***						
**Wild** **Australian**	0.533					
**Wild** **Indian**	0.496	0.290				
**Domesticated Indian**	0.808	0.812	0.565			
**Domesticated** **Tropical**	0.812	0.829	0.573	0.050		
**Philippines**	0.712	0.667	0.448	0.165	0.179	

**Table 4:** Pairwise estimates of Nei’s genetic distance (lower diagonal) and gene flow (Nm) values (upper diagonal) among wild and domesticated groups.

	Wild *scarabaeoide*	Wild Australian	Wild Indian	Domesticated Indian	Domesticated Tropical	Philippines
**Wild** ***scarabaeoides***		0.219	0.254	0.059	0.058	0.101
**Wild** **Australian**	0.349		0.611	0.058	0.052	0.125
**Wild** **Indian**	0.476	0.135		0.192	0.186	0.309
**Domesticated Indian**	1.578	0.661	0.414		4.705	1.264
**Domesticated** **Tropical**	1.574	0.662	0.416	0.004		1.148
**Philippines**	1.254	0.549	0.333	0.028	0.029	

## Discussion

Here we have investigated the genetic diversity and population structure of domesticated pigeonpea and its wild relatives in the genus *Cajanus*. Because the genotypes we studied represent both shallow sampling of widely diverged species as well as relatively deep sampling of the single cultivated species, we combined both phylogenetic and population genetic analyses. The data were sufficient to derive relationships that were simultaneously congruent with, and more detailed than, previous plastid and nuclear gene phylogenies (MT. Kassa, PhD Dissertation). Moreover, the results permit assignment of *C. cajanifolius* as the most probable progenitor species, and they allow us to infer the origin of modern cultivated pigeonpea from nested population bottlenecks, with an initial domestication in India and subsequent spread of cultivation to tropical regions beyond India.

Crop domestication is accompanied by genome-wide reduction in genetic diversity [1]. This reduction derives from a population bottleneck imposed during the founding of a new crop lineage [30] and subsequently due to selection on specific loci that confer agronomically important traits [31]. Bottleneck severity varies among crop species depending on the duration of domestication and number of domestication events. For example, several grasses have about two-thirds of the genetic diversity found in their wild relatives [32], and simulations reveal a more severe bottleneck for rice than maize [31], [33]. Previous studies using SSR [24] and DArT [25] markers detected a reduction in levels of genetic diversity in domesticated pigeonpea compared to wild relatives though the degree of a bottleneck effect was not quantified.

Here we quantify the reduction in genetic diversity, estimating that domesticated pigeonpea contains only ?25% of polymorphic loci present in the progenitor wild-India group. Only 62 markers detected variation among the domesticated *C. cajan* group (excluding the Philippines accessions) in comparison to 283 SNP markers that were polymorphic among the progenitor wild-India accessions. It is noteworthy that landraces (primitive cultivars) and improved (elite) cultivars that comprise the domesticated portion of our genotype panel (Table 3) contained similar levels of polymorphic SNPs, indicating that much of the diversity that survived through the incipient stages of domestication was retained in current day cultivars and breeding lines. Despite the genetically narrow base of pigeonpea, the cultigen is noted for high levels of morphological diversity. Thus, different genotypes are adapted for acceptable agronomic yield in both tropical and semi-arid regions of the world, as reflected in the eco-geographical variation in collection sites for accessions used in this study. Similar genetic bottleneck effects have also been observed in other crop species such as soybean [34], [35], sunflower [36], and lima beans [37].

Although there was no clear distinction between landraces and modern cultivars, domesticated genotypes were resolved into two sections based primarily on the results of Neighbor Joining and STRUCTURE analyses (Figures 5A and 5B). The subdivision reflects the geographical origin of the respective genotypes, further supporting the validity of the groups, with one lineage of Indian origin containing approximately twice the genetic diversity of a second lineage of tropical origin. Both of these populations are depauperate of genetic diversity, with low genetic differentiation and low genetic distance between them. Taken together, these results suggest that primary domestication occurred in India, with a more recent nested bottleneck associated with genotypes grown in tropical regions. We suggest that the genetic distinctiveness of the tropical and Indian subgroups within *C. cajan* likely derives from breeding for the geographically-wide but agro-climatically similar tropical regions versus semi-arid environments.

Although limited within-taxon sampling reduces our ability to assess genetic diversity in the wild species, we can still make preliminary assessments of diversity in the *Cajanus* species that are important members of the secondary gene pool. In particular, we note low diversity in the wild accessions collected from Australia. This situation is curious, because the wild-Australia group contains seven distinct taxonomic species, yet possesses less than one-third the polymorphism found in the taxonomically homogeneous *C. scarabaeoides* lineage of Indian origin. A majority of these Australian species are endemic to Australia and possess similar morphological characters (e.g. leaf shape, leaf and flower color and the growth habit) [25]. Australia has been designated as an important center of species diversity for *Cajanus* [38], but our results argue against this conclusion because genetic diversity was quite low among the seven species used in this analysis.

Several lines of evidence indicate that the Australian lineage is closely related to the lineage of *non-C. scarabaeoides* wild-India species, including the sister relationship of these two lineages in Parsimony analysis (see legend to Figure 2 for clarification) and their low genetic differentiation (Fst = 0.290) relative to other among group comparisons (Table 3). As noted above, the Australian lineage is genetically homogeneous, with polymorphism rates less than twice that observed in the domesticated-India pigeonpea. These results are consistent with recent introduction of *Cajanus* into Australia from India, with a corresponding genetic bottleneck. Could human migration have been a factor? It is likely that migrating humans carried seed for nutrition, if not planting. If so, then genetic drift and new climates would have had pronounced effects on the characteristics of even casually collected seed stocks; for example, Australia’s climate is highly varied and differing moisture regimes would likely drive rapid divergence in adaptive leaf traits without a requirement for corresponding genome-wide diversification. Such morphological diversification could explain the proliferation of species assignments based on morphometric criteria, despite correspondingly low genetic diversity.

Interestingly, genetic evidence suggests that humans may have colonized Australia by migration from the Indian subcontinent. A proposed but controversial early migration route includes movement from the Indian sub-continent to Australia in the late Pleistocene, i.e., >10,000 years ago [39], while a proposed more recent event corresponds to changes to the anthropological record in Australia around 5,000 to 3,000 years ago [40]. Although we have no evidence that humans either cultivated or carried pigeonpea along this migration route, the apparent origin of related *Cajanus* spp in India and the presence of a narrow genetic base of derived *Cajanus* species in Australia are consistent with this possibility. If true, then this “Australian-focused *Cajanus* bottleneck” was entirely independent of the recognized Indian-domestication, because the modern cultivated pigeonpea is genetically distinct from its Australian relatives.

Gene flow subsequent to initial domestication is also likely to have contributed to the character of cultivated pigeonpea, and the haplotypes of several accessions provided evidence of recent genetic admixture between wild and cultivated gene pools. The potential for gene flow is significant, as insect-aided natural out-crossing for pigeonpea may range up to 70% [41]–[45] and recently up to 17% natural out-crossing has been observed for wild species [44], with the highest out-crossing rate recorded for *C. lineatus*. Van der Maesen (personal communication) notes that multiplication of *C. lineatus* in experimental gardens is common practice in India and therefore the occurrence of spontaneous hybrids involving *C. lineatus* and nearby cultivated *C. cajan* should be expected and has been observed. Here we identified one highly admixed genome described as *C. lineatus*.

In addition to the above referenced *C. lineatus* accession, the highest rates of admixture from the wild-India population were observed for two Philippines accessions (ICP 12765 and ICP 10880), ICRISAT reference set accession (ICP 11975), and two accessions of *C. cajanifolius* (C7847 and ICPW 32) collected from the field. Of particular interest are two accessions of *C. cajan* (ICP 15629 and ICPW 29) that possess genomes that are predominantly domesticated, but with 5–10% membership of the wild-Indian group. *C. cajan* ICP 15629 and ICPW 29 served as parental lines to develop a stable cytoplasmic male sterility (CMS) system in pigeonpea. The CMS accession, ICP 2039A, was derived from an inter-specific hybrid of ICPW 29 and cultivar ICP 11501 [46]; we speculate that intentional hybridization and repeated backcrossing may have contaminated the genome of these parental accessions.

### Conclusions

This molecular diversity study corroborates the long held alpha-taxonomic hypothesis that *C. cajanifolius* is the most recent progenitor of cultivated pigeonpea and supports India as the most likely center of pigeonpea domestication. However, crop domestication is a progressive process that may involve both independent derivations within the range of the ancestral species (in the sense of Allaby et al. [47]) and hierarchical selection events that together span thousands of years and serve to adapt germplasm to diverse eco-geographical conditions. Moreover, when crops are grown in the vicinity of locally adapted wild species, there is great potential for both intentional and accidental genetic admixture, which would further impact allele content in the cultivated gene pool. Cultivated pigeonpea would be particularly prone to such admixture, given its significant out-crossing rates and documented cross-compatibility with local wild species. In the current analysis, we used population structure analysis and AMOVA to reveal genetic admixture between wild and cultivated genomes, suggesting the involvement of gene flow between wild and domesticated species. The wild gene pool of *Cajanus* contains not only high genetic diversity but also unique and rare alleles for agronomically important traits (e.g. trichomes of *C. scarabaeoides* for pod borer resistance; *C. platycarpus* has shown to be the only source of resistance to the P3 race of *Phytophthora* blight disease). Thus pigeonpea breeding and improvement programs would benefit from the continued and expanded use of this bounty of genetic diversity prevailing in the wild gene pool.

## Materials and Methods

### Plant Materials

As listed in Table S1, the 110 accessions of *C. cajan* and allied species used in this study derive from diverse environments in Asia, Australia, Africa and the Caribbean. With the exception of 12 wild accessions, which were acquired from the Western Australia Herbarium and were originally collected from the field in Australia, all other genotypes were obtained from the gene bank of the International Crops Research Institute for the Semi-Arid Tropics (ICRISAT) or from germplasm resources at Panjabrao Deshmukh Agricultural University (PDAU), University of Agricultural Sciences (UAS)-Bangalore, or UAS-Dharwad. Within the cultivated genotypes several accessions derive from core and mini-core accessions circumscribed by ICRISAT based on various morphological descriptors, and agro-morphology traits and SSR diversity data [48]–[50].

### Molecular Methods

Genotyping was based on a set of 752 SNPs discovered in a comparison of 1440 sequenced amplicons between *C. cajan* accession ICP 28 and *C. scarabaeoides* accession ICPW 94 (Table S2 and available at www.comparative-legumes.org/). Polymorphisms were identified by amplicon re-sequencing and sequence alignment involving *C. cajan* (ICP 28) and *C. scarabaeoides* (ICPW 94). The target sequences were a set of primarily single copy orthologous genes, whose orthology was inferred initially from legume EST data (i.e., the transcriptomes of *Medicago truncatula*, *Lotus japonicus* and *Glycine max*) and subsequently based on conserved genome location in a multi-species comparative genetic analysis (Penmetsa and Cook, unpublished data). Individual SNPs meeting assay design criteria, determined by Illumina Inc. using their proprietary Assay Design Tool, were converted to a 768 Illumina GoldenGate genotyping assay. Together these SNP assays survey biallelic states at 670 distinct genes. For purposes of genotyping, DNAs were extracted from the 110 *Cajanus* accessions using the Qiagen DNeAsy protocol using a Retch mixer mill, according to manufacturer’s instruction, and delivered to the UC Davis Genome Center DNA Technologies Core for analysis (http://dnatech.genomecenter.ucdavis.edu/). Allele calls were curated using the Illumina Beadstudio software package (Illumina, San Diego, CA, USA). To minimize the confounding effects of technical error, all SNP calls with more than 10% missing data were excluded from the analysis. Details on sequence context of SNPs, and the curated genotyping data (locus x genotype) are provided in Table S2.

### Data Analysis

As our genotypes included both diverged species and samples from the breeding pool where interbreeding and/or admixture occurs or has occurred often in the recent past, we took both phylogenetic and population genetic approaches to the data. We first used phylogenetic methods to distinguish the major groups, having a greater number of informative markers than previous studies. We then took the least well-resolved group of domesticated germplasm and most closely related wild species and analyzed it with population genetic approaches. Phylogenetic analyses were conducted using both the character based phylogenetic analysis of maximum parsimony and the distance-based analysis of Neighbor Joining. The maximum parsimony analysis was based on the complete data set of the concatenated SNPs at 670 loci and the analysis was performed using PAUP* 4.0b10 [51]. A full heuristic search was performed with 1000 random addition sequence replicates using Tree Bisection Reconnection (TBR) branch swapping. Clade support was evaluated through bootstrapping with 500 replicates using TBR branch swapping and the results were used to generate a consensus parsimony tree. To further deduce overall similarity among accessions and resolve relationships between individual genotypes, pairwise dissimilarity was calculated by simple matching according to the method of Saito and Nei [52]. The resulting dissimilarity matrix was used to derive a weighted Neighbor Joining tree [52] with 1000 bootstraps. This weighted Neighbor Joining analysis employs a likelihood-based criterion that models distance between genotypes as random variables that obey a Gaussian distribution [53]. The analysis was carried out with DARwin5 software [54].

Genetic structure was analyzed using the program STRUCTURE 2.1 [28], [29]. STRUCTURE assigns individual genotypes to a specified number of groups “K” based on membership coefficients calculated from the genotype data. The analysis was run from K = 1 to 10 using a burn-in period of 50,000 steps followed by 500,000 MCMC (Monte Carlo Markov Chain) replicates with 2 iterations, assuming admixture and correlated allele frequencies. Optimal K, which is adopted as the number of sub-populations from which the analyzed accessions derive, was determined using an *ad hoc* static ΔK based on the rate of change in the natural log probability of data between successive K values as described by Evanno and colleagues [55]. At optimal K, individual sub-populations were extracted and analyzed separately using STRUCTURE 2.1 to resolve additional genetic relationships.

Based on the output of STRUCTURE and principal coordinate analysis (PCoA) (see below), combined with phylogenetic inferences and common knowledge of geography and breeding history, we circumscribed 6 subgroups for further analysis. Analysis of Molecular Variance (AMOVA) was conducted, based on the hierarchical model and permutational procedures of Excoffier and colleagues [56], to assess the level of variation among these wild and domesticated groups. To avoid the potential confounding effects of admixture (revealed by STRUCTURE), we removed all admixed genotypes (except the Philippines accessions) and AMOVA was performed on 95 genotypes. Genetic variation within groups (Fct), variation within populations (Fst) and variation between populations within a group (Fsc), population polymorphism, and Nei’s Genetic distance and gene flow (Nm) were analyzed using GenAlEx v.6.3 [26] and Arlequin [57]. A principal coordinate analysis (PCoA) using GenAlEx v.6.3 was conducted to complement the output of the phylogenetic analyses, with the former being most sensitive to differences among groups and the later more sensitive to differences between closely related individuals [27].

## Supporting Information

Table S1 **Details of individual** ***Canajus*** **spp accessions.**
(DOCX)

Table S2 **SNP assay design and genoytpe data.**
(XLSX)

Table S3 **Ln of the probability and its variance for K from 1 to 10, provided as supporting information for** **Figure 4** **.**
(DOCX)

Table S4 **Ln of the probability and its variance for K from 1 to 5, provided as supporting information for** **Figure 5** **.**
(DOCX)
