# Supplementary material for: Genetic Patterns of Domestication in Pigeonpea (Cajanus cajan (L.) Millsp.) and Wild Cajanus Relatives
Source: PLoS One. 2012 Jun 22;7(6):e39563. doi: 10.1371/journal.pone.0039563 (PMC3382124; doi:10.1371/journal.pone.0039563)
Supplement: Table S1 — Details of individual Canajus spp accessions. (DOCX) [file pone.0039563.s001.docx]

| **Table S1. Description of *Cajanus* accessions** | | | | | | | |
| --- | --- | --- | --- | --- | --- | --- | --- |
| **Genotype**  **No**  **(Fig .4)** | **Genotype**  **name** | **Genetic**  **Status** | **Source of material** | **Origin** | **Accession** | **Species**  **Name** | **Genotype**  **No**  **(Fig. 5B)** |
| 1 | ICP 28-1 | cultivar | ICRISAT | India | Pusa Ageti | *C. cajan* | 19 |
| 2 | ICP 28-2 | cultivar | ICRISAT | India |  | *C. cajan* | 20 |
| 3 | ICPW 94-1 | Wild | ICRISAT | India |  | *C. scarabaeoides* |  |
| 4 | ICPW 94-2 | Wild | ICRISAT | India |  | *C. scarabaeoides* |  |
| 5 | C. scarabaeoides-C0651 | Wild | PERTH | Australia | 03340651 | *C. scarabaeoides* |  |
| 6 | C. lanceolatus-C7698 | Wild | PERTH | Australia | 05937698 | *C. lanceoltus* |  |
| 7 | C. reticulatus C7960 | Wild | PERTH | Australia | 06867960 | *C. reticulatus* |  |
| 8 | C. cinereus-C8603 | Wild | PERTH | Australia | 06698603 | *C. cinereus* |  |
| 9 | C. latisepalus C8898 | Wild | PERTH | Australia | 06508898 | *C. latisepalus* |  |
| 10 | C. cajan-C6364 | Natural | PERTH | Australia | 05146364 | *C. cajan* |  |
| 11 | C. cajan-C5225 | Natural | PERTH | Australia | 06225252 | *C. cajan* |  |
| 12 | C. hirtopilosus -C0828 | Wild | PERTH | Australia | 05430828 | *C. hirtopilosus* |  |
| 13 | C. pubescens -C0666 | Wild | PERTH | Australia | 07380666 | *C. pubescens* |  |
| 14 | C. reticulatus C7837 | Wild | vd Maesen | Australia | VDM 7837 | *C. reticulatus* |  |
| 15 | C. cajanifolius-C7847 | Wild | vd Maesen | India | VDM 7847 | *C. cajanifolius* |  |
| 16 | C. crassus-C7832 | Wild | vd Maesen | India | VDM 7832 | *C. crassus* |  |
| 17 | ICP 15756 | Wild | ICRISAT | Indonesia | KLM 786 | *C. scarabaeoides* |  |
| 18 | ICP 15644 | Wild | ICRISAT | India | NKR | *C. lineatus* |  |
| 19 | ICP 7035 | Cultivar | ICRISAT | India | Mosaic Res. | *C. cajan* | 40 |
| 20 | ICP 8863 | Cultivar | ICRISAT | India | ICWR-6 | *C. cajan* | 18 |
| 21 | ICP 12039 | Cultivar | ICRISAT | Unknown |  | *C. cajan* | 32 |
| 22 | ICP 12043 | Cultivar | ICRISAT | Unknown |  | *C. cajan* | 33 |
| 23 | ICP 22049 | Cultivar | ICRISAT | Unknown |  | *C. cajan* | 34 |
| 24 | ICPR 2438 | R-Line | ICRISAT | Unknown |  | *C. cajan* | 71 |
| 25 | ICPR 2447 | R-Line | ICRISAT | Unknown |  | *C. cajan* | 72 |
| 26 | ICPR 2463 | R-Line | ICRISAT | Unknown |  | *C. cajan* | 73 |
| 27 | ICPR 2671 | R-Line | ICRISAT | Unknown |  | *C. cajan* | 74 |
| 28 | ICPL 2 | Landrace | ICRISAT | Unknown |  | *C. cajan* | 44 |
| 29 | ICPL 332 | Landrace | ICRISAT | Unknown |  | *C. cajan* | 45 |
| 30 | ICPL 84023 | Landrace | ICRISAT | Unknown |  | *C. cajan* | 46 |
| 31 | ICPL 85010 | Landrace | ICRISAT | Unknown |  | *C. cajan* | 47 |
| 32 | ICPL 85030 | Landrace | ICRISAT | Unknown |  | *C. cajan* | 48 |
| 33 | ICPL 87091 | Landrace | ICRISAT | India | Early maturing | *C. cajan* | 49 |
| 34 | ICPL 87119 | Landrace | ICRISAT | Asha |  | *C. cajan* | 50 |
| 35 | ICPL 88034 | Landrace | ICRISAT | Unknown |  | *C. cajan* | 51 |
| 36 | ICPL 88039 | Landrace | ICRISAT | Unknown |  | *C. cajan* | 52 |
| 37 | ICPL 99050 | Landrace | ICRISAT | Unknown |  | *C. cajan* | 53 |
| 38 | ICPL 20096 | Landrace | ICRISAT | Unknown |  | *C. cajan* | 54 |
| 39 | ICPL 20097 | Landrace | ICRISAT | Unknown |  | *C. cajan* | 55 |
| 40 | ICPL 20102 | Landrace | ICRISAT | Unknown |  | *C. cajan* | 56 |
| 41 | ICPL 20108 | Landrace | ICRISAT | Unknown |  | *C. cajan* | 57 |
| 42 | TTB7 | cultivar | University of Agricultural Sciences (UAS), Banglore | Unknown |  | *C. cajan* | 21 |
| 43 | AKT8811 | cultivar | Dr. Panjabrao Deshmukh Agricultural University (PDAU), Akola | Unknown |  | *C. cajan* | 22 |
| 44 | TAT10 | cultivar | Dr. Panjabrao Deshmukh Agricultural University (PDAU), Akola | Unknown |  | *C. cajan* | 23 |
| 45 | BSMR736 | cultivar | Dr. Panjabrao Deshmukh Agricultural University (PDAU), Akola | Unknown |  | *C. cajan* | 24 |
| 46 | T.VISHAKA | cultivar | Dr. Panjabrao Deshmukh Agricultural University (PDAU), Akola | Unknown |  | *C. cajan* | 25 |
| 47 | C11 | cultivar | Dr. Panjabrao Deshmukh Agricultural University (PDAU), Akola | Unknown |  | *C. cajan* | 26 |
| 48 | G T 288 | cultivar | Dr. Panjabrao Deshmukh Agricultural University (PDAU), Akola | Unknown |  | *C. cajan* | 27 |
| 49 | GULLYL White | cultivar | University of Agricultural Sciences (UAS), Dharwad | Unknown |  | *C. cajan* | 28 |
| 50 | GULLYAL Red | cultivar | University of Agricultural Sciences (UAS), Dharwad | Unknown |  | *C. cajan* | 29 |
| 51 | GS1 | cultivar | University of Agricultural Sciences (UAS), Dharwad | Unknown |  | *C. cajan* | 30 |
| 52 | ICP 2376 |  | ICRISAT | India | RG 102; P 3888 | *C. cajan* | 41 |
| 53 | BSMR 736 | cultivar | ICRISAT | Unknown |  | *C. cajan* | 31 |
| 54 | C. sericeus | Wild | ICRISAT | Unknown |  | *C. Sericeus* |  |
| 55 | ICPW 12 | Wild | ICRISAT | Unknown |  | *C. latisepalus* |  |
| 56 | ICPW 46 | Wild | ICRISAT | Unknown |  | *C. lineatus* |  |
| 57 | ICPW 29 | Wild | ICRISAT | Unknown |  | *C. cajanifolius* |  |
| 58 | ICPW 68 | Wild | ICRISAT | Unknown |  | *C. paltycarpus* |  |
| 59 | ICPW 69 | Wild | ICRISAT | Unknown |  | *C. paltycarpus* |  |
| 60 | ICPW 94-3 | Wild | ICRISAT | India |  | *C. scarabaeoides* |  |
| 61 | ICPW 130 | Wild | ICRISAT | Unknown |  | *C. scarabaeoides* |  |
| 62 | ICP 49 | PI 394548 | ICRISAT | India |  | *C. cajan* |  |
| 63 | ICP 1071 | P 4578 | ICRISAT | India |  | *C. cajan* |  |
| 64 | ICP 1535 | Core | ICRISAT | India | P 3816/1-1 | *C. cajan* | 5 |
| 65 | ICP 4266 | Core | ICRISAT | India | P 631 | *C. cajan* | 6 |
| 66 | ICP 6523 | Core | ICRISAT | India |  | *C. cajan* | 7 |
| 67 | ICP 6933 | Core | ICRISAT | Trinidad | Code No. 3 | *C. cajan* | 16 |
| 68 | ICP 7337 | Reference | ICRISAT | India | ANM 16 | *C. cajan* | 63 |
| 69 | ICP 7409 |  | ICRISAT | India | ANM 79 | *C. cajan* | 42 |
| 70 | ICP 7782 | Core | ICRISAT | India |  | *C. cajan* | 8 |
| 71 | ICP 7941 | Core | ICRISAT | India |  | *C. cajan* | 9 |
| 72 | ICP 8095 |  | ICRISAT | India | ANM 450 | *C. cajan* | 43 |
| 73 | ICP 8242 | Reference | ICRISAT | India | PLA 332 | *C. cajan* | 64 |
| 74 | ICP 8265 |  | ICRISAT | Unknown |  | *C. cajan* | 35 |
| 75 | ICP 8817 | Reference | ICRISAT | India | Kuselghat 1 | *C. cajan* | 65 |
| 76 | ICP 9236 | Reference | ICRISAT | India | PI 394816 | *C. cajan* | 66 |
| 77 | ICP 10240 | PI 394590 | ICRISAT | India |  | *C. cajan* | 36 |
| 78 | ICP 10531 | PI 396259 | ICRISAT | India |  | *C. cajan* | 37 |
| 79 | ICP 10880 | PI 275 | ICRISAT | Philippines |  | *C. cajan* | 38 |
| 80 | ICP 10922 | T-2 (III-5) | ICRISAT | Australia |  | *C. cajan* | 39 |
| 81 | ICP 10963 | Core | ICRISAT | India | RPSP 580 | *C. cajan* | 10 |
| 82 | ICP 11246 | Core | ICRISAT | Unknown |  | *C. cajan* | 3 |
| 83 | ICP 11543 |  | ICRISAT | Unknown | ICPL 87 | *C. cajan* | 75 |
| 84 | ICP 11754 | Reference | ICRISAT | Unknown | ICPL 304 | *C. cajan* | 70 |
| 85 | ICP 11975 |  | ICRISAT | Philippines | D-0 Type | *C. cajan* | 60 |
| 86 | ICP 12079 | Core | ICRISAT | Tanzania |  | *C. cajan* | 14 |
| 87 | ICP 12094 | Core | ICRISAT | Tanzania | PR 5474 | *C. cajan* | 15 |
| 88 | ICP 12765 | Reference | ICRISAT | Philippines | PR 5302-4 | *C. cajan* | 69 |
| 89 | ICP 12977 | Core | ICRISAT | India | PR 6088-1 | *C. cajan* | 11 |
| 90 | ICP 13004 | Reference | ICRISAT | India | PR 6109-1 | *C. cajan* | 67 |
| 91 | ICP 13799 | Core | ICRISAT | Trinidad | PR 6499 | *C. cajan* | 17 |
| 92 | ICP 14126 | Reference | ICRISAT | Jamaica | PR 6692 | *C. cajan* | 68 |
| 93 | ICP 14153 | Core | ICRISAT | Brazil | EEI 85137 | *C. cajan* | 1 |
| 94 | ICP 14389 | Core | ICRISAT | C. Africa | AK 288 | *C. cajan* | 2 |
| 95 | ICP 14444 | Minicore | ICRISAT | Unknown | ICPL 85021 | *C. cajan* | 58 |
| 96 | ICP 14471 | Minicore | ICRISAT | Unknown | ICPL 86014 | *C. cajan* | 59 |
| 97 | ICP 14524 | Core | ICRISAT | India | MRP 146 | *C. cajan* | 12 |
| 98 | ICP 14770 | Reference | ICRISAT | Unknown | ICPL 332 | *C. cajan* | 62 |
| 99 | ICP 15454 | Core | ICRISAT | Nigeria | PRAN 21 | *C. cajan* | 13 |
| 100 | ICP 16198 | Core | ICRISAT | Unknown |  | *C. cajan* | 4 |
| 101 | ICP 16235 | Reference | ICRISAT | Unknown | ICPL 92042 | *C. cajan* | 61 |
| 102 | ICP 15614 | Wild | ICRISAT | India | JM 2337 | *C. albicans* |  |
| 103 | ICP 15627 | Wild | ICRISAT | India | ICPW 026 | *C. albicans* |  |
| 104 | ICP 15629 | Wild | ICRISAT | India | ICPW 028 | *C. cajanifolius* |  |
| 105 | ICP 15632 | Wild | ICRISAT | India | ICPW 031 | *C. cajanifolius* |  |
| 106 | ICP 15762 | Wild | ICRISAT | Unknown |  | *C. sericeus* |  |
| 107 | ICP 15882 | Wild | ICRISAT | India | ICPW 281 | *C. scarabaeoides* |  |
| 108 | ICP 15665 | Wild | ICRISAT | India | ICPW 064 | *C. paltycarpus* |  |
| 109 | ICP 15747 | Wild | ICRISAT | India | ICPW 146 | *C. scarabaeoides* |  |
| 110 | ICP W94-4 | Wild | ICRISAT | India |  | *C. scarabaeoides* |  |
