# Supplementary material for: Genetic Patterns of Domestication in Pigeonpea (Cajanus cajan (L.) Millsp.) and Wild Cajanus Relatives
Source: PLoS One. 2012 Jun 22;7(6):e39563. doi: 10.1371/journal.pone.0039563 (PMC3382124; doi:10.1371/journal.pone.0039563)
Supplement: Table S3 — Ln of the probability and its variance for K from 1 to 10, provided as supporting information for Figure 4 . (DOCX) [file pone.0039563.s003.docx]

| **K** | **Ln P(D)** | **Var [Ln P(D)]** |
| --- | --- | --- |
| 1 | -60704 | 351.7 |
| 2 | -23763.6 | 8031.5 |
| 3 | -17531 | 8626.7 |
| 4 | -15519.2 | 5057.1 |
| 5 | -14950.2 | 8389.8 |
| 6 | -41902.8 | 62805.1 |
| 7 | -29443.5 | 38155.2 |
| 8 | -86595.5 | 152144.5 |
| 9 | -42162.7 | 65209.7 |
| 10 | -29710.8 | 39323.2 |

Table S3. Ln of the probability and its variance for K from 1 to 10, provided as supporting information for Figure 4.
