# Supplementary material for: Genetic Patterns of Domestication in Pigeonpea (Cajanus cajan (L.) Millsp.) and Wild Cajanus Relatives
Source: PLoS One. 2012 Jun 22;7(6):e39563. doi: 10.1371/journal.pone.0039563 (PMC3382124; doi:10.1371/journal.pone.0039563)
Supplement: Table S4 — Ln of the probability and its variance for K from 1 to 5, provided as supporting information for Figure 5 . (DOCX) [file pone.0039563.s004.docx]

Table S4. Ln of the probability and its variance for K from 1 to 5, provided as supporting information for Figure 5.

| **K** | **Ln P(D)** | **Var [Ln P(D)]** |
| --- | --- | --- |
| 1 | -5766.3 | 94 |
| 2 | -4782.1 | 192.8 |
| 3 | -4859 | 961.9 |
| 4 | -5158.6 | 1940 |
| 5 | -4555.7 | 829.3 |
